# Pain Behaviour Scale (PaBS): An Exploratory Study of Reliability and Construct Validity in a Chronic Low Back Pain Population

**DOI:** 10.1155/2019/2508019

**Published:** 2019-02-03

**Authors:** Dalyah M. Alamam, Andrew Leaver, Niamh Moloney, Hana I. Alsobayel, Ghada Alashaikh, Martin G. Mackey

**Affiliations:** ^1^Arthritis and Musculoskeletal Research Group, Faculty of Health Sciences, The University of Sydney, 75 East Street, Lidcombe, NSW 2151, Australia; ^2^Department of Rehabilitation Sciences, College of Applied Medical Sciences, King Saud University, Riyadh 12371, Saudi Arabia; ^3^Department of Health Professions, Faculty of Medicine and Health Sciences, Macquarie University, 75 Talavera Road, NSW 2109, Australia; ^4^THRIVE Physiotherapy, Guernsey, Channel Islands, UK; ^5^Department of Physiotherapy, Prince Sultan Military Medical City, Riyadh 12233, Saudi Arabia

## Abstract

**Objectives:**

To examine the interrater and intrarater reliability and construct validity of the Pain Behaviour Scale during standard physical performance tests in people with chronic low back pain and to confirm the test-retest reliability of the physical performance tests in this population. The Pain Behaviour Scale (PaBS) is an observational scale that was recently designed to uniquely measure both the presence and severity of observed pain behaviours.

**Methods:**

Twenty-two participants with chronic low back pain were observed during performance of five physical performance tests by two raters. Pain behaviours were assessed using the Pain Behaviour Scale. The Visual Analogue Scale and Modified Oswestry Disability Index were used to measure pain and disability, respectively. Descriptive statistics were used to report demographic features of participants. Reliability was analyzed using ICCs. Rater agreement was analyzed using the weighted Cohen's kappa. Correlations between PaBS, self-reported measures, and physical performance tests were calculated using Pearson's product-moment correlations.

**Results:**

The PaBS demonstrated excellent interrater (ICC_2_,_1_ = 1.0, 95% CI: 0.9 to 1.0) and intrarater (ICC_3,1 _= 0.9, 95% CI: 0.8 to 1.0) reliability. Component physical performance tests (i.e., time and distance) demonstrated good test-retest (0.6–1.0) reliability. Perfect agreement in the reporting of pain behaviours was found (95–100%). Correlations between pain behaviour severity and pain intensity (*r* = 0.6) and disability (*r* = 0.6) were moderate. Moderate correlations were found between pain behaviours and physical performance tests in sit to stand (*r* = 0.5), trunk flexion (*r* = 0.4), timed up and go (*r* = 0.4), and 50-foot walk (*r* = 0.4).

**Conclusion:**

The Pain Behaviour Scale is a valid and reliable tool for measuring the presence and severity of pain behaviour, and the physical performance tests are reliable tests.

## 1. Introduction

Chronic low back pain (CLBP) is one of the leading causes of disability that is often described as nonspecific and is recognized as having multiple contributing factors [1, 2]. It affects most people at some point in their lives with recurrent episodes common [3]. It has a significant impact on an individual's psychological health, social life, and physical performance [2, 4].

Pain has been defined as “an unpleasant sensory and emotional experience associated with actual or potential tissue damage or described in terms of such damage” [5]. This definition indicates that the perception of pain is a result of a complex interaction of physical, physiological, behavioural, and sociocultural factors [6, 7] and may be derived from the cognitive-emotional factors which influence the pain experience [6]. An individual's experience of pain may be outwardly expressed by demonstration of certain aberrant behaviours, with visible or audible responses indicative of discomfort or suffering such as grimacing and sighing [8, 9]. Such pain-related behaviours are commonly seen in musculoskeletal clinical practice in people with conditions such as osteoarthritis [10] and CLBP [11, 12]. Therefore, reporting perceived pain intensity on a Visual Analogue Scale and demonstrating pain-related behaviours are two different ways in which people may communicate their suffering in a clinical setting [6]. Further, pain-related behaviours such as guarding can cause some individuals to indicate that they have more pain [13] or are associated with pain-related psychological factors such as fear avoidance behaviour or catastrophizing [14]. Previous research has shown that pain catastrophizing and pain-related fear of movement may account for significant variance in the experience of pain and pain behaviour [14] and may feed the cycle of fear avoidance [15]. A number of studies have also shown a positive association between pain behaviour and disability among people with low back pain (LBP). Therefore, quantification of pain behaviour should be considered during clinical assessment [15–18].

Clinicians' interpretation of these behaviours is an important part of a multidimensional patient assessment and treatment. There is growing recognition of the importance of integrating both physical and psychosocial factors in clinical assessments [19]. Indeed, a multidimensional assessment that includes assessment of pain-related functional behaviours is the cornerstone to contemporary interventions for back pain such as cognitive functional therapy [7]. Assessment of pain behaviours may help health professionals to predict the severity of a low back pain (LBP) condition [20, 21] and may initiate a conversation between the clinician and patient around factors such as their thoughts, feelings, beliefs, and physical variables influencing observed behaviours. Subsequently, such clinical reasoning may influence approaches to intervention such as cognitive behavioural therapy (CBT). For example, frequent and severe pain-related behaviours have been shown to change following CBT [22].

In previous research, special attention has been directed towards rating the presence or the frequency of pain behaviours [23]. Similarly, previous observational studies in people with LBP evaluated the frequency of pain behaviour [24–28]. It is argued that measuring frequency alone only captures one aspect of the patient's clinical presentation. Measuring frequency alone may not discriminate between patients who exhibit different degrees of pain-behaviour severity [29]. Therefore, a pain-behaviour measure that better reflects the whole pain experience would be a valuable clinical tool for practitioners. Several attempts to measure pain behaviour using different constructs and in different settings have also been conducted [30, 31]. However, to our knowledge, no similar assessment tool exists to measure the presence and severity of commonly observed chronic pain behaviours.

Assessment tools that are reliable, reproducible, and practical are imperative in good clinical practice [32]. In people with CLBP, common pain-related behaviours include grimacing, sighing, breath-holding, guarding, and antalgic gait [14, 24, 33, 34]. In previous studies, behavioural assessments of people with LBP have been conducted while participants performed standard physical examination procedures such as measurement of strength and range of motion [24, 25, 27, 34, 35]. However, this approach has been criticized for not evaluating the patient during functional task performance [25]. In previous studies, the presence of pain-related behaviours was usually informally assessed by the clinician through direct observation or videotaping [25, 27, 34–36]. Videotaping is less practical as it requires equipment and can be time consuming [25]. Evaluation of behaviours by direct observation has been reported previously, but only the presence or absence of pain behaviours has been described, and found to be reliable. However, the degree or severity of the observed pain behaviour has not previously been considered [16, 24, 25, 27, 35]. This latter feature is an important clinical finding to evaluate the degree to which an individual is affected and as a reassessment measure.

Simmonds et al. [37] demonstrated that a suite of timed functional tests commonly used in rehabilitation medicine were able to discriminate people with CLBP from normal healthy people. This suite of tests included repeated trunk flexion, repeated sit to stand, timed up and go, loaded reach, and 50-foot walk tests [37]. The reliability of this suite of tests has been established and as a simple, low-tech measure, has good utility for assessment of CLBP [37]. Because this suite of tests includes a range of reproducible functional activities, its potential to act as a platform for testing a new scale for assessing pain behaviours during functional movement was apparent. Although the reliability of this suite of functional tests has been examined previously, we wanted to ensure that the tests were reliable among the study population. As the stability of the test scores indicate that the tests could be used as a basis for testing the Pain Behaviour Scale (PaBS) in this population. We developed PaBS to rate the presence and severity of observable pain behaviours during a suite of functional tests performed by CLBP patients. The PaBS has potential clinical application to assess pain behaviours during regular physical examination. To our knowledge, no similar assessment tool exists to quantify both the presence and severity of pain behaviours during functional movement. We, therefore, hypothesized that the PaBS would have acceptable interrater and intrarater reliability and construct validity. We also hypothesized that the physical performance tests would have acceptable test-retest reliability.

The purpose of this study was to examine the intrarater and interrater reliability as well as the construct validity of the PaBS and to confirm the test-retest reliability of the physical performance tests in this population.

## 2. Materials and Methods

### 2.1. Setting

Participants were recruited from amongst a cohort of people with LBP who were participating in a multicenter study that was investigating cultural aspects of back pain in Saudi Arabia. Male and female study participants who consecutively attended a physiotherapy outpatient clinic in Riyadh, Saudi Arabia, between January and March 2016, were invited to participate. Eligible participants were provided with information about the study at the time of their initial appointment. Potential participants were screened for inclusion criteria and provided written informed consent prior to testing.

### 2.2. Participants

Based on previous studies [25, 38–40], we were aiming to recruit at least 20 participants as a sufficient sample for the aim of the study. Volunteers were included if they were Saudi citizens over 18 years of age, with nonspecific CLBP of more than three months' duration. Exclusion criteria were (1) clinical features of serious pathology (e.g., malignancy, infection, inflammatory disorders or fracture, and spinal cord or cauda equina syndrome); (2) specific pathologies such as lumbar radiculopathy; (3) a history of back surgery; (4) pregnancy; or (5) being incapable of completing written questionnaires in Arabic.

### 2.3. Procedure

Participants provided baseline demographic and clinical data and completed a series of questionnaires upon entry to this study. Participants were asked to perform a standardized sequence of physical performance tests. These tests are commonly used in physiotherapy assessment and are reflective of normal daily functional activities [37]. The procedure was derived from a series of timed functional tests published by Simmonds et al., to which we added measures for scoring pain-behaviour severity [37].

### 2.4. Measurements

#### 2.4.1. Pain Behaviour Scale (PaBS)

The PaBS was developed to record the presence and severity of pain behaviours exhibited during the physical performance tests. The specific pain behaviours assessed were sighing, breath-holding, grimacing, guarding, rubbing, and antalgic gait [33]. Assessment of antalgic gait was only possible during the timed up and go and 50 foot walk tests. These pain behaviours are commonly exhibited by people with significant pain [33]. The PaBS consists of a 4-point scale, ranging from “None” (i.e., no observed behaviour) to “Severe” (i.e., marked pain behaviour). Two measures were obtained from the scale: (1) the presence or absence of each behaviour and (2) a total score of the severity of the overall pain behaviours. Regarding the criteria for determining severity, we evaluated the severity of the observed pain behaviour (i.e., in terms of how intense or marked the behaviour appeared). The total score of severity (0–15) was determined by summing the individual ratings of severity for the pain behaviours observed for each test with higher total scores indicating greater severity of observed pain behaviours (Appendix A). The scale was administered during the performance of physical tests which takes 10–15 minutes.

#### 2.4.2. Physical Performance Tests

The PaBS was obtained during the performance of a standardized sequence of the original physical performance tests as described by Simmonds et al. (1998) which comprised the following measurement components:

1. Repeated trunk flexion: The time taken in seconds (s) for the participant to flex to the limit of their range of motion and return to the upright position as fast as tolerable 10 times.
2. Repeated sit to stand: The time taken (s) to rise to standing and return to sitting 5 times as quickly as possible.
3. Timed up and go: The time taken (s) to rise from the seated position, stand and walk forward to a line 3 metres away, turn, walk back to the chair and sit down.
4. Loaded reach: The participant was asked to stand next to the wall holding a weight by their side not exceeding 5% of body mass and then reach forward at shoulder height with the load. The maximum reach distance (cm) was recorded.
5. 50-foot walk: The time taken (s) to walk 25 feet, turn around, and walk back to the starting position, as fast as possible.

Each participant took part in two test sessions (session 1 and session 2) that were separated by one week and occurred before they commenced treatment. The physical performance tests were performed in both testing sessions. Other physical performance tests as described by Simmonds et al., such as the unloaded forward reach test, did not distinguish LBP and control groups [37]. This test is less physically challenging compared to the loaded reach test and therefore was excluded. In addition, as the 50-foot walk and 5 minute walk tests are highly correlated [37], the latter test was not included.

#### 2.4.3. Self-Report Measurements

Demographic characteristics (age, gender, marital status, education, smoking status, and work-related information) were collected using a standardized form. Self-reported disability and pain intensity were assessed using the Modified Oswestry Disability Index (MODI) and the Visual Analogue Scale (VAS), respectively [41, 42]. The MODI consists of 8 items related to physical function. Scores are calculated out of 100, with scores >21 indicating moderate disability [4, 43]. Arabic translation of the MODI has excellent intraobserver reliability (ICC: 0.99) and good construct validity [42]. The VAS for pain consists of 10-cm line, the left end labeled “No pain” and the right end labeled “Severe pain” [44].

### 2.5. Raters

Data were collected by two physiotherapists. Both physiotherapists had a minimum seven years' clinical experience in managing musculoskeletal conditions. Each rater was trained in the use of PaBS. Training of rater 1 (R1) consisted of three days' training with a senior physiotherapist in a pain clinic including review of the scale and the definitions for each observation as well as practical experience using the PaBS on people with chronic pain attending the pain clinic. Rater 1 subsequently trained rater 2 (R2) over a similar period.

### 2.6. Testing Procedure

Independent observations were performed by R1 and R2. Participants were informed that they would perform a standardized sequence of physical performance tests. A trained research assistant provided participants with verbal instructions for each physical performance test. The participant performed the tests, and the research assistant recorded the time taken and/or the distance for each test, respectively. Raters were located in front of the participant during the performance of each test. Each rater independently and simultaneously recorded which pain behaviours were observed and the perceived severity of the observed pain behaviours of each test. In addition, an overall score of pain severity was determined at the completion of all tests. Raters were blinded to each other's results throughout the test session [37]. Further, for the assessment of intrarater reliability, R1 was blinded to the previous ratings during the second test session. As all participants recruited had CLBP, raters were not blinded to participants' condition. Ethical approval for this study was obtained from the Human Research Ethics Committee of the University of Sydney, Australia (2015/771).

### 2.7. Statistical Analysis

Data entry was completed by an independent researcher who was unaware of the study purpose. Data were normally distributed, and descriptive statistics were used to report demographic and clinical features of participants. Mean, standard deviation (SD), and range values were reported for continuous data, and frequencies and percent for were reported for categorical data. Differences between pain intensity, disability level, PaBS scores, and the component physical performance tests (i.e., time and distance) were analyzed using paired *t*-tests. Obtaining results from paired *t*-tests was important, as this could influence the test-retest reliability results, e.g., if the patient's condition had changed and intrarater reliability was poor [45]. Intrarater and interrater reliability of the PaBS were analyzed using intraclass correlation coefficients (ICC) with 95% confidence intervals (CIs) reported. For calculation of intrarater reliability, the ICC_3,1_ was used, accounting for measures taken over two test sessions. For interrater reliability (rating of R1 and R2 at session 1), ICC_2,1_ was used [37, 46, 47]. These analyses were performed for each test separately and then for the total scale score.

Test-retest reliability for the component physical performance tests was determined using ICC_1,1_. Standard errors of measurement were calculated [37, 47]. ICC values were interpreted according to guidelines established by Shrout and Fleiss, where values > 0.75 indicate excellent reliability, 0.6–0.75 good reliability, 0.4–0.59 fair reliability, and <0.4 poor reliability [46–48]. A minimum value of 0.70 is considered acceptable before advocating this tool be used in practice [49].

The level of agreement between raters in reporting the presence or absence of pain behaviours during each physical performance test was analyzed using the weighted Cohen's kappa test and percentage of agreement [50, 51]. According to Cohen, correlation coefficients of ≤0.10 are “small,” those of ≤0.30 are “medium,” those of ≤0.50 are “large,” and those of >0.50 are “very large” [52].

In order to assess the construct validity, correlations between PaBS, self-reported pain, disability, and components of physical performance tests (i.e., time and distance) were calculated using Pearson's product-moment correlation [53]. The correlation coefficient was interpreted as follows: excellent ≥0.75, moderate 0.50–0.75, fair 0.25–0.50, and poor relationship <0.25 [46, 47]. The correlation coefficients were interpreted according to the constructs being tested. A correlation greater than 0.5 is considered acceptable between the PaBS scores and conceptually related constructs such as pain intensity and disability [54]. All statistical tests were performed using SPSS 22 (SPSS, Inc., Chicago, IL, USA).

## 3. Results

Twenty-two participants were included with a mean age of 32 years, half of whom were female. Most of the participants were professional workers such as healthcare providers (45%). There were no significant differences in the levels of reported pain (*t* (21) = −0.83, (*p*=0.42)) and disability (*t* (21) = 0.90, (*p*=0.93)) between the first and second test sessions. The characteristics of participants, including self-reported measures (pain and disability), are presented in Appendix B.

A summary of the participants' component physical performance tests, PaBS total score, and PaBS scores for each physical performance test are presented in Table 1. There were no significant differences in the component physical performance tests or PaBS scores between first and second sessions.

A summary of the frequency of the presence of different pain-related behaviours as rated by R1 and R2, for each physical performance test is presented in Table 2. The most frequently observed pain behaviours were grimacing during the timed up and go test, while breath-holding and guarding were most notable during the loaded reach test. Sighing was more frequently observed during the trunk flexion test.

Intrarater and interrater reliability results for the PaBS are presented in Table 3. The overall PaBS score demonstrated excellent interrater (ICC = 1.0 (95% CI: 0.9 to 1.0)) and intrarater (ICC = 0.9 (95% CI: 0.8 to 1.0)) reliability. In addition, component physical performance tests (time and distance) demonstrated good to excellent test-retest reliability (ICC = 0.7–1.0 (95% CI: 0.3 to 1.0)). Internal consistency could not be established for this scale because it was not possible to calculate a total score from the physical performance test (time and distance) components [55].

Agreement between raters for the presence of individual pain behaviours for each test was between 95 and 100% with the exception of sighing during the timed up and go test (91% agreement), grimacing during the loaded reach test (86% agreement), and guarding during the trunk flexion test (81% agreement). Weighted kappa scores revealed nearly perfect consistency in reporting the presence/absence of pain behaviours during the physical performance tests (Table 4).

Moderate correlations were identified between the pain behaviour severity score and both pain intensity (*r* = 0.6, *p* < 0.01) and disability scores (*r* = 0.6, *p* < 0.01). Moderate correlations were found between pain behaviour severity and physical performance time measures for sit to stand (*r* = 0.5, *p* < 0.01), trunk flexion (*r* = 0.4, *p* < 0.01), timed up and go (*r* = 0.4, *p* < 0.01), and 50-foot walk (*r* = 0.4, *p* < 0.01). A poor correlation was found between the pain behaviour severity score and reach distance in the loaded reach test (*r* = 0.2, *p* < 0.01).

## 4. Discussion

This study tested selected psychometric properties of the PaBS, which assessed the presence and severity of pain behaviours during standardized functional tests in people with CLBP. The PaBS demonstrated excellent intrarater and interrater reliability and acceptable construct validity. The PaBS is a simple, easily administered instrument that has potential clinical application to assess and monitor pain behaviours in people with CLBP, and possibly in other patient groups. To our knowledge, no similar instrument is currently available to clinicians for this purpose.

Our results demonstrate that it is possible to obtain highly reliable measurements of the presence and severity of pain behaviours, characteristics which would be highly relevant to clinicians in assessment, and treatment of patients presenting with LBP. The severity of observable pain behaviours using the PaBS revealed excellent interrater (1.0) and intrarater reliability (0.9) (Table 3). Fair reliability was only found for the loaded reach test; however, this outcome did not influence the overall test reliability of the scale. The finding of slightly lower intrarater reliability of the loaded reach test might be due to the effect of time with a one-week interval between the two test sessions during which the participants' condition may have changed in some way. In our study, pain intensity measures (0–10 on VAS) were slightly higher in session 2 (4.8) than those in session 1 (4.4) (Appendix B); however, the change score was not statistically significant. In addition, if the functional tests were pain provoking initially, it is possible that the participants might have anticipated a repeated experience of pain during the second test session, possibly affecting results. There was near-perfect agreement between raters about whether individual pain behaviours were present or absent during each test. These results suggest that PaBS is stable across time when being used for people with CLBP by trained raters.

The PaBS provides a means of behavioural assessment of patients during physical performance tests. Physical performance tests are commonly used in assessment of people with CLBP, and normative values have been published and can be compared with related values in people with CLBP [25, 37, 56]. We found that most of the component physical performance tests showed excellent consistency between the repeated measurements. However, repeated sit to stand and trunk flexion tests in this study showed slightly less consistency between test sessions. Raters might find this challenging to observe consistently. It is also possible that the repeated movements in these two physical performance tests could be considered physically challenging [57]; therefore, the performance could differ for individuals between sessions.

Pain behaviours in this study were more frequently observed during the loaded reach and repeated trunk flexion tests than the other physical performance tests. These functional movements are commonly affected in people with LBP and may be associated with actual or feared exacerbations of pain or increased physical challenge [58]. Therefore, the finding of more frequent pain behaviours during these activities is consistent with clinical practice [58, 59] and with previous research findings [37]. The most frequently observed behaviours in previous studies [34, 60], as well as in this study, were grimacing, guarding, breath-holding, and sighing. It has been reported that individuals exhibit behaviours frequently as a method of communication, as a way to avoid unpleasant feelings or to maintain their control during activities [61, 62]. Knowledge of the presence of pain-related behaviours may in turn facilitate treatment to reach an effective outcome [7], particularly when evaluated as part of a multidimensional assessment where the influence of other physical and psychological factors is concurrently assessed [7, 19].

A moderate association between pain behaviour severity with pain intensity and with disability was found in this study (*r* = 0.6 for both). Participants who reported their pain and/or disability as being more severe demonstrated greater pain behaviours. The association between subjective rating of pain and disability and physical examination of observed behaviours enhances the relevance of assessing pain behaviours during clinical assessment. Further research is required to evaluate whether changes in pain, disability, and pain behaviours also correlate in response to treatment. Furthermore, in this study, PaBS demonstrated moderate correlations with most individual component physical performance tests assessed. This relationship supports the construct validity of the scale as a clinical tool in people with CLBP. These results give insight into the broader construct of pain behaviours, as the PaBS assesses two different dimensions of pain behaviour (i.e., presence and severity) and thus supports the pain behaviour construct.

The main strength of this study is the ability to administer a reliable tool in a clinical setting during the performance of physical tests that are commonly used during the assessment of people with CLBP and are reflective of normal daily functional activities. The findings of this study must be considered within the context of some limitations. The study was conducted in a small population, which may need further evaluation in a larger population to generalise outcomes to a broader population. The second rater was trained by the first rater which may impact the interrater reliability of the scale and may improve the level of agreement between raters. Future studies could ask novice raters to read an instruction manual of the pain behavioural scale and then rate a patient's pain behaviours independently by videotaped pain-behaviour assessment. We did not collect longitudinal data in order to examine the sensitivity of the scale to change over time. Longitudinal studies to examine how pain behaviours change over time and whether they can predict pain and disability would support the usefulness of the scale.

## 5. Conclusion

In conclusion, pain-related functional behaviours are important to assess as part of a multidimensional assessment in clinical practice. The PaBS was developed as a tool to improve comprehensive assessment of pain behaviours during the performance of functional tests commonly used in the assessment of people with LBP. The tool demonstrated excellent intrarater and interrater reliability and acceptable construct validity. The PaBS is a simple, easily administered instrument that has potential clinical application to assess and monitor pain behaviours in people with CLBP and possibly other patient groups.

## Figures and Tables

**Table 1:** Components of physical performance tests and PaBS descriptive data as rated by raters R1 and R2.

	First session	Second session	Mean difference (95% CI)	*t*	*p* value
	Mean (SD)^*∗*^	Mean (SD)			
Trunk flexion (s)^*∗*^	23.5 (9.5)	26.6 (18.5)	−3 (−8.0 to 1.8)	−1.31	0.20
Sit to stand (s)	13.7 (3.5)	14.0 (4.6)	−0.2 (−1.8 to 1.7)	−0.32	0.76
Timed up and go (s)	9.0 (2.4)	9.1 (2.2)	−0.08 (−0.7 to 0.5)	−0.27	0.79
Loaded reach (cm)	23.2 (7.3)	23.0 (7.4)	0.09 (−1.8 to 1.9)	0.09	0.92
50-foot walk (s)	13.0 (4.2)	13.0 (3.8)	0.01 (−0.5 to 0.5)	0.02	0.98
Trunk flexion					
R1^*∗*^	1.0 (1.0)	1.0 (1.0)			
R2	1.0 (0.8)	NA^*∗*^	−0.1 (−0.3 to 0.07)	−1.4	0.19
Sit to stand					
R1	0.5 (0.7)	0.5 (0.9)			
R2	0.5 (0.7)	NA	0 (−0.2 to 0.2)	0.00	1.00
Timed up and go					
R1	0.3 (0.6)	0.5 (0.9)			
R2	0.4 (0.7)	NA	−0.1 (−0.3 to .0)	−1.8	0.08
Loaded reach					
R1	1.2 (0.5)	1.1 (0.5)			
R2	1.2 (0.5)	NA	0.1 (−0.1 to 0.3)	0.81	0.43
50-foot walk					
R1	0.5 (0.8)	0.4 (0.8)			
R2	0.5 (0.8)	NA	0.1 (−0.1 to 0.3)	1.4	0.19
Pain behaviour					
Total PaBS1^*∗*^	3.5 (2.8)	3.6 (3.4)	−0.05 (−0.6 to 0.6)	−0.16	0.88

s, second; R1, rater 1; R2, rater 2; PaBS1, Pain Behaviour Scale scores as rated by R1 in the first and second sessions; NA, not applicable.

**Table 2:** Frequency of the presence of different pain related behaviours during physical performance tests rated by R1 and R2.

Test	Sighing (yes)	Breath-hold (yes)	Grimacing (yes)	Guarding (yes)	Rubbing (yes)	Antalgic gait (yes)
Repeated trunk flexion, *n* (%)						
R1	13 (59.1)	7 (31.8)	6 (27.3)	5 (22.7)	5 (22.7)	NA
R2	14 (63.6)	8 (36.4)	6 (27.3)	3 (13.6)	5 (22.7)

Repeated sit-to-stand, *n* (%)						
R1	3 (13.6)	6 (27.3)	1 (4.5)	4 (18.2)	1 (4.5)	NA
R2	4 (18.2)	5 (22.7)	1 (4.5)	4 (18.2)	1 (4.5)

Timed up and go, *n* (%)						
R1	4 (18.2)	1 (4.5)	0	1 (4.5)	2 (9.1)	4 (18.2)
R2	4 (18.2)	0	0	1 (4.5)	2 (9.1)	4 (18.2)

Loaded reach, *n* (%)						
R1	8 (36.4)	18 (81.8)	6 (27.3)	17 (77.3)	1 (4.5)	NA
R2	7 (31.8)	18 (81.8)	5 (22.7)	17 (77.3)	0

50-foot walk, *n* (%)						
R1	4 (18.2)	3 (13.6)	1 (4.5)	2 (9.1)	1 (4.5)	4 (18.2)
R2	4 (18.2)	2 (9.1)	2 (9.1)	1 (4.5)	1 (4.5)	4 (18.2)

NA, not applicable.

**Table 3:** Results of interrater and intrarater reliability for the severity of pain behaviour and test-retest reliability of component physical performance tests.

Test	Interrater reliability for PaBS	Intrarater reliability for PaBS	Test-retest of component physical performance tests
	ICC (95% CI)	ICC (95% CI)	ICC (95% CI)
Total score	1.0 (0.9 to 1.0)	0.9 (0.8 to 1.0)	NA
Trunk flexion (s)	0.9 (0.8 to 1.0)	0.9 (0.7 to 0.9)	0.7 (0.4 to 0.9)
Sit to stand (s)	1.0 (0.9 to 1.0)	0.8 (0.5 to 0.9)	0.6 (0.3 to 0.8)
Timed up and go (s)	0.9 (0.9 to 1.0)	0.9 (0.8 to 1.0)	0.8 (0.6 to 0.9)
Loaded reach (cm)	0.8 (0.5 to 0.9)	0.4 (0.3 to 0.7)	0.8 (0.6 to 0.9)
50-foot walk (s)	0.9 (0.8 to 1.0)	0.8 (0.6 to 0.9)	1.0 (0.9 to 1.0)

All ICC values were significant at *p* < 0.05.

**Table 4:** Agreement between raters about the presence of pain behaviours during the physical performance tests.

Test	Weighted kappa	*p* value
Trunk flexion	0.9	*p* < 0.001
Sit to stand	1.0	*p* < 0.001
Timed up and go	0.9	*p* < 0.001
Loaded reach	0.8	*p* < 0.001
50-foot walk	0.9	*p* < 0.001

All *p* values were significant at *p* < 0.001.

**Table 5:** 

Height ________		Weight _________				

Test	Time	Pain behaviours				□ Sighing^1^
Repeated trunk flexion (×10)	sec	0	1	2	3	□ Breath-hold
		None	Mild	Moderate	Severe	□ Grimace
						□ Guarding
						□ Rubbing


Test	Time	Pain behaviours				□ Sighing
Repeated sit-to-stand (×5)	sec	0	1	2	3	□ Breath-hold
		None	Mild	Moderate	Severe	□ Grimace
						□ Guarding
						□ Rubbing


Test	Time	Pain behaviours				□ Sighing
Timed up and go	sec	0	1	2	3	□ Breath-hold
		None	Mild	Moderate	Severe	□ Grimace
						□ Guarding
						□ Rubbing
						□ Antalgic gait


Test	Reach	Pain behaviours				□ Sighing
Loaded reach	cm	0	1	2	3	□ Breath-hold
		None	Mild	Moderate	Severe	□ Grimace
						□ Guarding
						□ Rubbing


Test	Time	Pain behaviours				□ Sighing
50-foot walk	sec	0	1	2	3	□ Breath-hold
		None	Mild	Moderate	Severe	□ Grimace
						□ Guarding
						□ Rubbing
						□ Antalgic gait

^1^Sighing: a clear exhalation accompanied by a rise and fall of the shoulders. Breath-hold: sharp intake and hold of the breath. Grimacing: a clear facial expression of pain that may include a furrowed brow, tightened lips, and clenched teeth. Guarding: abnormal and stiff movement during shifting. Rubbing: rubbing the affected body part to relieve pain. Antalgic gait: impaired walking due to pain.

**Table 6:** 

Scores	Description
0	The participant has a relaxed face and normal breath pattern and moves easily and freely with normal gait.
1	The participant shows one of the following: minor wrinkles in the forehead and mild breath-holds or sighs, demonstrates mild guarding, and touching the affected part or mild gait impairment.
2	The participant shows one of the following: obvious wrinkles in the forehead, clear breath-holds, or sighs, appears hesitant to move, and demonstrates guarding, rubbing the affected part for at least 3 seconds, or an obvious impaired gait.
3	The participant shows one of the following: a clear facial expression of pain that may include a furrowed brow, tightened lips, and clenched teeth. The patient demonstrates a sharp breath-holding or frequent audible sighs and demonstrates guarding and stiff movement during movement and rubbing of the body part for more than 3 seconds or clear impaired gait.

**Table 7:** 

Age, mean (SD^*∗*^)	32.3 (12.1)	
Range	21–65	

Female, *n* (%)	12 (54.0)	

Married, *n* (%)	11 (50.0)	

Education, *n* (%)		
** **No formal education	1 (4.5)	
** **High school	5 (22.7)	
** **Diploma certificate	1 (4.5)	
** **University degree	15 (68.2)	

Nonsmoker, *n* (%)	20 (90.0)	

Occupation, *n* (%)		
** **Office worker	4 (18.0)	All computer users
** **Professional worker	10 (45.0)	All professional workers such as healthcare providers
** **Other	8 (36.0)	All nonworkers (i.e., house wives and students)

Pain^*∗*^, mean (SD)		
** **First session	4.4 (2.6)	Mean difference between sessions (95% CI)
** **Second session	4.8 (2.5)	−0.4 (−1.5 to 0.6)

Disability^*∗*^, mean (SD)		
** **First session	19 (9.9)	Mean difference between sessions (95% CI)
** **Second session	19 (11.5)	11 (−2.5 to 2.8)

SD, standard deviation. ^*∗*^Pain was measured using the Visual Analogue Scale (0–10), and disability was measured using the Modified Oswestry Disability Index (0–50).

## Data Availability

The data used to support the findings of this study are restricted by the Human Research Ethics Committee of the University of Sydney, Australia (2015/771), in order to protect patient privacy. Data are available from the corresponding author Dalyah Alamam via email for researchers who meet the criteria for access to confidential data.

## Appendix

### A. The Pain Behaviour Scale (PaBS)

The Pain Behaviour Scale shown in Table 5.

Interpretation of the scores [11] (Table 6).

### B. Participant Characteristics

Participant characteristics shown in Table 7.
